# An Overview of Current Approaches and Challenges to the Control of Endemic Infectious Cattle Diseases in Albania

**DOI:** 10.3389/fvets.2021.671873

**Published:** 2021-07-14

**Authors:** Xhelil Koleci, Ali Lilo, Sotiraq Papa, Keti Margariti, Annika van Roon, Inge Santman-Berends, Gerdien van Schaik, Jaka Jakob Hodnik, Sam Strain, Maria Guelbenzu-Gonzalo, Esa Karalliu

**Affiliations:** ^1^Department of Veterinary Public Health, Faculty of Veterinary Medicine, Agricultural University of Tirana, Tirana, Albania; ^2^Veterinary & Animal Welfare Sector, Ministry of Agriculture & Rural Development, Tirana, Albania; ^3^Unit Farm Animal Health, Department of Population Health Sciences, Faculty of Veterinary Medicine, Utrecht University, Utrecht, Netherlands; ^4^Department of Epidemiology, Royal Gezondheidsdienst voor, Deventer, Netherlands; ^5^Clinic for Reproduction and Large Animals, Veterinary Faculty, University of Ljubljana, Ljubljana, Slovenia; ^6^Animal Health and Welfare Northern Ireland, Dungannon, United Kingdom; ^7^Animal Health Ireland, Carrick-On-Shannon, Ireland

**Keywords:** cattle diseases, control programme, disease freedom, Standardizing Output-based surveillance to control Non-regulated Diseases of cattle in the EU, Albania

## Abstract

Agriculture is an important production sector in Albania that makes a significant contribution to the gross domestic product (GDP) and employment. The livestock sector contributes more than half of the agricultural GDP. The Albanian cattle population represents 50% of the total livestock units and accounts for 85% of the national milk production, the rest being supplied by small ruminants. Cattle productivity, health and welfare are hindered by infectious diseases, some of which are also transmissible to humans (zoonosis). The aim of this manuscript is to provide an overview of the control of selected regulated and non-EU regulated cattle diseases in Albania and to highlight specific challenges for the Albanian cattle industry. The most important infectious cattle diseases in Albania for which national control and eradication strategies are in place are bovine brucellosis, bovine tuberculosis, and anthrax, which are all zoonotic. Additionally, lumpy skin disease recently emerged in the Balkan region and is currently subject to controls. Most of the available funds and European Union support are allocated to the control of EU regulated zoonotic diseases. For control of non-EU regulated cattle diseases, no funds are available resulting in the lack of national control programmes (CPs). Based on research, clinical investigations and laboratory results, several non-EU regulated cattle infectious diseases appear endemic in Albanian dairy farms. While no national CPs exist for any of them, regional initiatives are available on a voluntary basis to control infectious bovine rhinotracheitis and bovine viral diarrhea. In the voluntary CPs, there is no monitored requirement to prove disease freedom of purchased animals and to re-evaluate the herd's free status after the introduction of animals into a herd. Data on animal movements that are routinely collected could potentially be used to control the risk of purchase, but quality needs to be further improved to increase its usefulness in disease CPs. This overview aims to collate existing information on the CPs implemented in Albania and to evaluate these to highlight gaps and threats in disease control, as well as opportunities and strengths through a SWOT (Strengths, Weaknesses, Opportunities, and Threats) analysis, with the goal of providing a framework for the future implementation of animal disease control measures in Albania.

## Introduction

Albania is an Eastern European Country in which agriculture, and the cattle sector in particular, play an important role, contributing substantially to the economy and employment opportunities. Albania is in the process of approximating and harmonizing its legislation with the European Union (EU) and seeks to increase livestock production, entrepreneurship, competitiveness, and improve the animal health status of Albanian livestock. Currently, in Albania, there are several national control programmes (CPs) for certain zoonotic infectious diseases of cattle namely bovine brucellosis, bovine tuberculosis and lumpy skin disease. However, there are no public nor private national CPs in place for non–EU regulated cattle diseases except anthrax disease.

A project called Standardizing Output-based surveillance to control Non-regulated Diseases of cattle in the EU (SOUND control), supported by the European Union has members from 33 countries including Albania. The overall aim of SOUND control is to explore and support the development of transparent methods that enable comparison of outputs of surveillance, control or eradication programmes of non-regulated cattle disease CPs in the EU. Within the first work package (WP1), a list of 24 non-EU-regulated (Supplementary Table 3) diseases for which at least one European country has a CP was published by SOUND control (1). In Albania, out of the 24 diseases five have never been detected, enzootic bovine leucosis occurs sporadically, and 11 diseases are endemic of which four are controlled to some degree (Supplementary Material). The aim of this manuscript is to provide an overview of the control measures in place for regulated and non-EU-regulated cattle diseases in Albania and to present a Strengths, Weaknesses, Opportunities and Threats (SWOT) analysis of the specific challenges for the Albanian cattle industry.

### The Cattle Industry and Disease Control in Albania

Albania is located in the Eastern part of Europe and has an approximate territory of 28,000 km^2^ and a population of 2.8 million people. The population density is 97 inhabitants per square km (2). The agriculture sector is a major contributor to the country's economy with about one-fifth of the gross domestic product (3). About 46% of the population lives in rural areas, and 650,000 people are employed in the private agricultural sector. Twenty-four percent of Albania's surface is arable, while 16% is pasture and 36% is forest (2). The total number of cattle in 2019 was 415,609, 11% less compared to 2018. The average cattle density is 40 cattle per km^2^ of agricultural land but varies substantially between regions (Supplementary Table 1) (ranging from 17 in Gjirokastër to 71 cattle 78 in Lezhë regions). The average dairy herd size is 2.6 cows (Supplementary Table 2) and only 1.1% of all farms have more than 11 cows (2).

**Figure 1:**
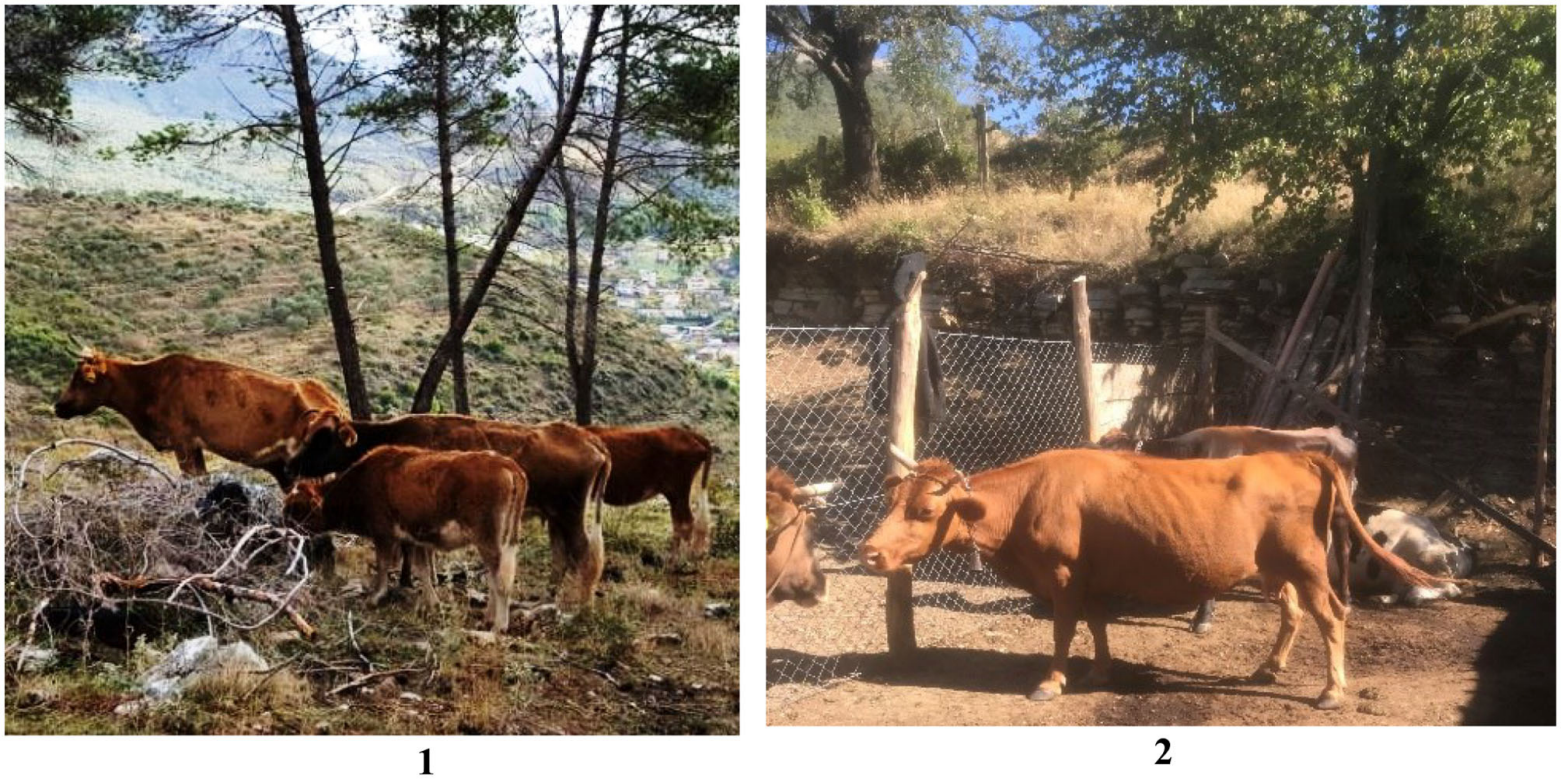
Typical local cattle breed in Southern Albania (1) used for meat production and cross bred cattle kept in Northern Albania (2) for meat and milk production.

The majority of Albanian cattle herds are composed of local breeds, known as Albanian Shorthorn Cattle (4) and crossbreeds (Figure 1) with a small number of dairy breed cattle imported from Germany, the Netherlands, Italy, Denmark, and Ireland. Most of the imported cows are Holstein breed, followed by Jersey, Bruna Alpina, and Simmental cattle (5).

Traditionally, cattle are managed using a combination of indoor and outdoor rearing. In lowland areas where pure breeds predominate, cattle are often reared indoors, with limited access to pasture. In contrast, in hills and mountain areas, local and crossbreed cattle are managed both indoor and outdoor, and often sheep and goats are reared at the same family farm (Supplementary Table 2). In Albania, there are comparatively few beef herds located mainly in the southern part of the country.

Dairy farms are relatively small and face several challenges including low milk prices, the high price of animal feed and supplements, farmland fragmentation, limited access to land irrigation, and bureaucratic procedures to obtain limited subsidies.

According to Albanian veterinary law, all imported cattle must be quarantined for 21 days, clinically examined, and screened for highly transmissible OIE listed diseases (6). However, in general, cattle farmers do not apply strict biosecurity measures to prevent disease introduction and there is a need to increase the farmers' awareness of the role of biosecurity in disease control. The veterinary law and ministry regulations (6) list the diseases that must be controlled using active surveillance and monitoring programmes, which are updated annually. The dedicated CPs are devoted to zoonotic diseases, and emergent and transboundary animal diseases of high priority in Albania (6).

## Materials

We reviewed the scientific literature, government guidelines, research institutions bulletins and experts' personal communications on the topics of cattle disease control programmes in Albania to identify cattle diseases programs, gaps and challenges. We categorized the points according to the SWOT matrix.

## Results

The cattle industry is the most important livestock industry in Albania; however, it is not well-developed, very extensive and faces a variety of factors that impede disease control. Table 1 shows some of the strengths, weaknesses, opportunities, and threats of cattle disease control in Albania.

**Table 1:** SWOT analyses considering cattle infectious disease control programmes in Albania.

**Strengths**	**Weaknesses**
**National and public level**: A clear chain of command within the State Veterinary Service. The availability of a national livestock and veterinary information system (RUDA). Valuable experience gained from applying strategic programmes for control of major zoonotic diseases. Human and laboratory capacities of the National Reference Laboratory. Continuous education and staff exchange programmes at national and international level. An Albanian veterinary faculty. Small farms have a higher animal welfare. **Farm level:** Traditional experience and successful family farms. Increasing use of artificial insemination and improving of local cattle breeds. Small herds. Periodical collection of bulk milk samples.	**National and public level**: Limited control and documentation of animal movements at both national and farm level. Limited animal identification and registration. Trade at livestock markets without disease control. Lack of calves for fattening. Lack of farm specialization. Lack of economically driven production. Lack of export of live cattle and dairy products. Limited research studies on the prevalence and incidence of endemic bovine diseases. Lack of investments in the cattle industry. Low number of government veterinary staff in central level. Government funds dedicated to only four diseases: bovine brucellosis, bovine tuberculosis, anthrax, and lumpy skin disease. Bureaucratic procedures to obtain limited financial support to farmers. Low level of organization of the farmer's associations. Limited data routinely recorded and stored centrally. Mandatory quarantine not enforced or applied. **Farm level:** Small and fragmented farmland. Use of communal pasture for herds with unknown disease status. High age of farmers and limited engagement of younger generations. Limited access to veterinary services in rural areas. Lack of farmer perception of infectious diseases and their impact in human health, herds efficiency and competitiveness. Application of limited biosecurity measures on cattle farms. Limited farmers' knowledge and compliance with legal requirements.
**Opportunities**	**Threats**
**National or public level**:Increasing demand for animal food products, alongside the development of agritourism. Increasing global human population and with it the demand for high value products. Collaboration with the scientific community in EU countries to gain coherent knowledge and expertise (such as SOUND control). Education of staff in the EU (Erasmus+, Horizon 2020, COST). EU funding for supporting Albania Veterinary Services. Export of animal products when zoonotic diseases are under control. Development tourism activities. **Farm level:** Increase farmer awareness for the importance of biosecurity in disease control. Organic animal products.	**National or public level:** Global warming may hamper cattle production. New emerging diseases. Difficulty in implementing new EU animal health regulations. Emigration. Lack of incentive for young generation. Societal pressures to reduce farm animal product consumption due to perceived health benefits and farm associated greenhouse gas emissions. The high average age of farmers. **Farm level**: Limited availability of alternative financial resources. Few younger people engage in the industry due to a lack of livestock enterprise competitiveness and increasing emigration. High cost of dairy and beef cattle products and lack of competitiveness with regional and European products.

### Strengths

The strengths of the cattle industry in Albania when focusing on infectious disease control are listed in Table 1. Imported pure-breed cattle and artificial insemination have contributed to changes in cattle management. Artificial insemination has allowed improved milk and meat yield productivity through accelerated genetic selection. Studies have shown that large herd size is a risk factor for several cattle diseases (7–9). To the best of the authors' knowledge, the most economically efficient farms in Albania are family herds, which are small (herds up to 20 milking cows), run by family members only, and use their own land. The small farms also have a higher animal welfare.

The new amendments of Albanian Veterinary Law aim to provide a clear chain of command within the State Veterinary Service making coordination and collaboration more effective. The national livestock and veterinary information system (RUDA system) is established and serves as an important information platform to integrate information from many areas within the veterinary domain. RUDA does provide resources to develop and support scientific and risk-based sampling for surveillance and animal disease control activities.

Strategic programmes for the progressive control and eradication of priority animal diseases and major zoonoses provided the opportunity to gain knowledge and skills, which could be used for controlling other cattle diseases. The national reference laboratory staff at the Food Safety and Veterinary Institute receive ongoing training and most of them hold advanced degrees.

The staff of the State Veterinary Service attend training events and workshops organized by international organizations/projects/other stakeholders to acquire knowledge and skills needed in the veterinary field.

### Weaknesses

The size of farms is small, with an average of 1.2 ha (2), which do not support large scale cattle management systems and are not profitable due to the small herd sizes (2). The small herds, however, tend to also rear other animal species and consequently are at risk of pathogen spread between different farm animal species.

It is difficult for beef herds to form fattening groups of calves of equal age due to the small herd size and therefore calves from Bulgaria and North Macedonia are imported. The health status of these calves, however, is not checked before their introduction into the herd and despite mandatory quarantine by law, this is usually not applied or enforced.

The so-called “beef” production herds are of particular interest in terms of their risk of spreading infectious diseases between herds and regions. Often, these farms practice summer mountain grazing. When the weather conditions in spring improve, cattle are moved to mountain pastures where they are kept exclusively outside and stay there until late autumn when they return to the “winter” holding. Breeding in these animals is natural and seasonal. The calving season is in early spring in “winter” holdings where biosecurity and hygiene conditions are usually poor. Milk is used for feeding calves and cows are either not milked or only milked for household consumption. Calves are usually slaughtered at about 6 months of age.

The trading of cattle is very frequent. Cattle are commonly bought and sold at livestock markets, where there are usually no disease control measures, or directly from farm to farm without any animal movement recording at the national level. The RUDA system serves as an important information platform to integrate information from many areas within the veterinary domain. However, RUDA is not updated regularly with valid and timely animal movement data. The State Veterinary Service staffing at the central level is too low. The identification of all domestic animals is not available yet in the RUDA system. Some modules are currently missing (i.e., pigs modules). There is a language barrier for use of on-line resources. The complex government tendering process often results in vaccines or other consumables arriving late with negative impacts on animal health programmes with seasonal variables. Regional veterinary laboratories are no longer available, and so all samples are sent directly to the national reference laboratory.

Poor collaboration between the main stakeholders is an obstacle, e.g., official collaborative efforts with Public Health to provide timely reporting of zoonotic diseases. The presence of endemic zoonotic diseases is an obstacle for animal and food of animal origin export. In many rural areas it is difficult to provide veterinary service due to the poor infrastructure. The high cost of investment in cattle industry and lack of livestock enterprise competitiveness are factors in the decrease of both the number and size of cattle farms. In general terms, there is a lack of data from well-designed research studies for many of the non–regulated cattle diseases, which makes it difficult to design appropriate CPs.

The average age of farmers is increasing with fewer younger people engaging in the industry. While farm business sustainability is dependent on disease control, the application of disease control and eradication programmes is costly, disincentivizing farmers to participate.

### Opportunities

The geographic position of Albania is favorable for agrotourism and cattle industry development, with an increasing demand for safe meat and dairy products. The recent positive trend of tourism development opens the prospect to market organic regional products.

Albania is an official candidate for accession to the European Union. This provides an opportunity for using EU support and development funds for capacity building and collaboration between the scientific community in European countries to gain knowledge (such as SOUND control) and share experiences as well as facilitating farmer training to increase their awareness of the importance of biosecurity in disease control.

### Threats

The circulation of endemic cattle diseases is a serious obstacle to the export of dairy products and live animals and contributes to a lack of interest in investing in the livestock industry by private initiatives. Biosecurity measures that prevent the mixing of animals from different herds are not in place i.e., animals from different herds share common pasture, roads, and water sources. Additionally, the boundaries between farms, if they exist, are often inadequate to prevent animal mixing and nose-to-nose contact.

Government funds are dedicated to only four diseases: bovine brucellosis, bovine tuberculosis, anthrax, and lumpy skin disease. This causes difficulty in allocating funds for the remaining important cattle diseases. The perception of farmers of the impact of non-zoonotic infectious diseases on herd efficiency and competitiveness is poor, inhibiting participation in voluntary disease CPs. In addition, the level of application of biosecurity measures is very limited. Bureaucratic procedures to provide limited subsidies to farmers and the overall lack of financial resources interferes with interest in private initiatives to invest in the livestock industry. The frequent change of leadership at the central and regional veterinary levels further diminishes technical independence and expertise.

Lack of incentives to invest in livestock sector and high emigration rate of labor force represent a significant challenge for the future development of cattle industry and implementing disease CPs.

In addition, importing cheap dairy and meat products from international markets is impacting the local industry as they compete with local animal origin products.

Likewise, farmer associations are poorly organized and ineffective leading to a limited engagement with endemic disease control from the Albanian farming industry.

## Discussion and Conclusion

The aim of this paper was to describe the current approaches to regulated and non-regulated cattle diseases in Albania, including those that are subject to control and those that are not. We identified the most important gaps and factors that hamper non-EU regulated cattle disease control and eradication programmes. The cattle industry is the most important livestock industry in Albania. However, it is not well-developed, very extensive, and faces significant challenges to achieve disease control. The major infectious diseases of cattle in Albania are bovine brucellosis, bovine tuberculosis, anthrax, and lumpy skin disease (10). These diseases are all regulated and are a priority for Albanian veterinary services. Government and EU projects support these control and eradication strategies, but a free status has not yet been achieved for any of these diseases. The uncertain health status for these diseases hampers the export of dairy products and live animals. Most of the available government funds are dedicated to controlling these four diseases, diverting funds from initiatives for controlling cattle diseases with no or only limited regulation at the European Union level.

Several non-EU-regulated cattle diseases are present with differing prevalence (11). However, no national CPs are currently in place for these infections, except Anthrax. A range of factors interferes with the application of national control strategies for these diseases such as limited financial resources, poor development of the cattle industry, farmer knowledge and perception of disease, the low level of organization of farmer's associations, and a lack of well-designed studies and reliable information on infection prevalence and incidence of certain diseases.

Out of 24 non-EU regulated cattle diseases that are included in the SOUND control project, 11 are endemic in Albania, five have never been reported, one occurs sporadically and for the remaining seven no data is available (Supplementary Table 3). There is a national CP in place to control anthrax, which is supported by the government and private initiatives at the regional level are in place for controlling IBR and BVD. These programs are not well-designed, need to be improved, monitored, updated, and supported for proper implementation (12). More research efforts need to be focused on these diseases in order to provide the scientific base for better CP design and to justify the allocation of appropriate funds.

Periodically collected bulk milk samples that are used for bovine brucellosis surveillance may provide a cost-effective opportunity to monitor several of these diseases (13).

Control and eradication of non-EU regulated diseases should also be supported (government, farmers association, laboratory, and academic community) to increase the health and welfare of cattle and to decrease disease-associated losses and antimicrobial usage.

The present study has limitations given the sparsity of available literature (particularly in English) and data on endemic diseases within Albania. In addition, we know that SWOT analyses have their own limitations use for assessing cattle disease control programmes. However, this manuscript presents the first overview of the current situation regarding disease control in cattle in Albania in which all available information was combined. It highlights the gaps in Albanian cattle disease control and can serve as a basis for further studies and implementation of disease control measures in Albania.

## Data Availability Statement

The original contributions presented in the study are included in the article/Supplementary Material, further inquiries can be directed to the corresponding author/s.

## Author Contributions

All authors listed have made a substantial, direct and intellectual contribution to the work, and approved it for publication.

## Conflict of Interest

The authors declare that the research was conducted in the absence of any commercial or financial relationships that could be construed as a potential conflict of interest.
