# Supplementary material for: An Overview of Current Approaches and Challenges to the Control of Endemic Infectious Cattle Diseases in Albania
Source: Front Vet Sci. 2021 Jul 14;8:671873. doi: 10.3389/fvets.2021.671873 (PMC8317491; doi:10.3389/fvets.2021.671873)
Supplement: Supplementary file 1 [file Data_Sheet_1.docx]

# Supplementary Material

## Regulated cattle disease programmes running in Albania.

The National CPs for cattle infectious diseases currently running in Albania are for Lumpy Skin Disease (LSD), bovine tuberculosis (bTB), bovine brucellosis (BB), and anthrax. The latter covers all susceptible farm animals, including cattle.

# Lumpy Skin Disease

Lumpy Skin Disease (LSD) is an economically important acute viral disease of cattle, transmitted by indirect and direct routes characterized by high morbidity and varying degrees of case fatality, especially in naïve cattle population (EFSA, 2017).

LSD was first reported in Albania in the mid-summer of 2016. It was probably introduced into the country from North Macedonia (by cattle movement and/or vectors) as part of the epizootic wave that started in that region in 2015 (Mercier et al, 2018). The national CP is based on animal movement restriction, import ban of live animals, and hides from certain countries, awareness campaigns, clinical surveillance, and mass vaccination. The cattle vaccination started right after the introduction of infection, and within two weeks, approximately 59% of cattle were vaccinated. The mass vaccination campaigns were implemented by the state veterinary service with the support of the EU LSD vaccine bank. However, the available vaccine was insufficient to cover the entire cattle population at that time, as unvaccinated cattle were scattered around the country and new outbreaks were recorded on a daily basis. The second vaccination phase ran from December 2016 to January 2017 and 67% of the cattle population was vaccinated (EFSA, 2017). The standard molecular testing and monitoring system that is in place shows that the vaccination was effective in preventing new LSD outbreaks (EFSA, 2018). The vaccination of all cattle > 3 months of age has been performed annually since 2017 and passive clinical surveillance is in place.

# Bovine tuberculosis

Bovine tuberculosis is a chronic bacterial disease of animals and humans caused by *Mycobacterium bovis*. The usual route of infection is by aerosol exposure, but ingestion of contaminated milk and other material may also cause infection. Tuberculosis may be localized without apparent clinical signs but can also progress to clinical disease which may include emaciation, lethargy, weakness, anorexia, low-grade fever, and pneumonia with a chronic, moist cough. The infected animal becomes progressively weaker and eventually dies (Quinn et al, 2011).

In 2018, the Albanian veterinary services initiated a bovine tuberculosis control programme (bTBCP) based on active surveillance in dairy herds with more than 10 animals using the Single Intradermal Test. (Ministry^[[1]](#footnote-1)^, 2019c). Positive cases are confirmed using the comparative tuberculin skin test. Where there are confirmed positive cases, a range of measures is taken including compulsory slaughter, movement restrictions, biosecurity investigations, follow-up testing (including associated high-risk herds such as neighbours or herds traded with), cleaning, and disinfection. A serological surveillance revealed 1.4% of animals testing positive (Koni *et al*, 2016). Next-Generation Sequencing on the *M. bovis* isolates from bTB positive animals showed that both *M. bovis* and *M. caprae* are circulating in the cattle population (Koleci Xh., personal communication).

Although the initial surveillance results suggested that bTB was not a substantial animal health problem in larger dairy herds i.e. herds with more than 10 animals, the disease tends to cluster and may cause severe losses in specific herds or geographical areas. Moreover, the disease situation is not completely clear in smaller herds and this will require further investigation before the full national strategy is defined. Proposed measures for the bTB CP are the continuation of surveillance and related measures in larger dairy herds. A compulsory element of the current programme is slaughterhouse surveillance which aims to identify infected animals (Ministry, 2019c).

Therefore, as with other disease control programmes, the effective control of bTB will require the parallel implementation of a range of measures that includes: a reliable identification and registration (I&R) system, animal movement controls, inspection and monitoring capacity, adequate laboratory capacity, on-farm cleaning and disinfection, financial and human resources and a fully accessible electronic database.

# Bovine brucellosis

Bovine brucellosis (BB) is a zoonotic bacterial disease, primarily caused by *Brucella abortus*. It causes abortion in cows, orchitis, and epididymitis in male animals, and human brucellosis. In addition, cattle are susceptible to *B. melitensis* and *B. suis* (Quinn et al, 2011).

In Albania, *B. melitensis* and *B. abortus* circulate in both large and small ruminants. The first human case of brucellosis was detected in Albania in 1925 (Mersinaj et al, 2013); however, a study based on Brucella DNA detection carried out in human bones in southern Albania suggested that brucellosis has been endemic since at least the Middle Ages (Mutolo et al, 2012). Cattle are susceptible to both *B. melitensis* and *B. abortus*, and so there is a need for specific CPs in all susceptible species in order to control and eradicate the disease (Quinn et al, 2011).

The national brucellosis CP in small ruminants started in 2012 when the first mass vaccination campaign was implemented (Ministry^[[2]](#footnote-2)^, 2019b). The strategy had a significant impact in reducing abortion cases in cattle and human cases. The number of human brucellosis cases dropped from 442 in 2012 to 160 in 2015 and 70 in 2018 (Alla, Bino, 2016, Ministry, 2019b). In addition, there was a significant decrease of *Brucella melitensis* isolates from aborted cattle fetuses. In 2008, the most prevalent pathogen of cattle abortion outbreaks was *B. melitensis*, compared to 2016, where *B. melitensis* was isolated from only one positive cow (Ferro et al, 2020). The first national programme to control bovine brucellosis (BB) was adopted in March 2016. The BB CP consisted of a series of actions towards the progressive control of the disease including active surveillance (screening all dairy farms and confirming positive cases at the herd and individual level, elimination of positive animals, and farmer compensation), passive surveillance (reporting abortion cases and follow up) and control measures in both infected and uninfected herds. Given the small size of herds, a more comprehensive and structured approach, aligned to European Union regulations, was adopted in these holdings (Ministry, 2019b). Active surveillance based on repeated quarterly bulk milk testing was introduced in herds of 21 or more cattle. Individual animal testing was conducted in all positive herds using screening tests, after which the standard control measures included slaughtering of positive and in-contact animals and cleaning and disinfection of the premises. Supplementary measures, including enforcement of passive reporting and traceability, were also implemented by veterinary services. In 2017, the CP expanded to herds with 11-20 cattle, which were included in the active surveillance scheme. The serological results of BB surveillance in 2018 indicated a 2% herd – prevalence (Ministry, 2019b), which suggests an overall low herd prevalence. A serological survey done in 38 randomly selected beef herds in southern Albania in late 2017, suggested however a different epidemiological situation in this sub-population of animals. These results indicated a very high overall herd-prevalence of 55% (95% CI 40% - 71%) and within-herd seroprevalence in infected herds (45%). Bacteriological isolation and typing of the causal organism from specimens collected from 16 slaughtered seropositive animals confirmed the presence of *B. abortus* (Ferro et al, 2020). In 2019, another serological survey in 120 small herds of cattle (3 to 9 animals per herd), in the Lushnja district, suggested no infection was detected (Ministry, 2019b)

Despite the successful implementation of a range of control measures, that reduced abortions and

human cases of brucellosis, we identified several gaps that must be addressed. These include a lack of farmer awareness related to the importance of the disease, its zoonotic risk, and their roles and responsibilities according to the Albanian veterinary law. Further, non-compliance with bovine animal identification and registration regulations is an issue resulting in a general absence of effective cattle movement control.

Field investigations indicated that unauthorized movement of cattle and buying animals of unknown health status were the predominant sources of brucellosis infection in previously Brucella-free holdings (Ferro et al, 2020, Ministry, 2019b). Other challenges include under-reporting of abortion cases (low number of samples submitted) as well as a lack of laboratory investigation (Ministry, 2019b).

Control of brucellosis is a multidisciplinary activity involving a range of public institutions, in particular, the public health authority and the authority responsible for food safety; their contributions are essential for effective disease control. The Ministry of Agriculture plays a lead role in the coordination of the activities towards achieving the goal of control and eradication. The highest priority is the identification and adoption of the most appropriate control approach for the local epidemiological conditions. The epidemiological situation in commercial dairy herds seems to be favorable as the herd prevalence was 2.84% in 2016 and decreased to 1.99% in 2018 (Ministry, 2019). However effective control requires the full enforcement of the control and preventive measures, which are necessary to minimize the time to eradicate and subsequently to keep the bovine population free of the infection.

Given the very high brucellosis prevalence in beef herds, the only applicable control approach is mass vaccination. The encouraging trend of the decreasing number of human cases achieved by mass vaccination has to be maintained and further improved by strict enforcement of veterinary public health measures (Alla&Bino, 2014, 2015, 2016, Ministry, 2019b). The successful implementation of the current brucellosis control strategy in small ruminants, and the extension of the CP to control bovine brucellosis in dairy and beef herds will create favorable conditions for Albania to achieve brucellosis-free status. Achieving a brucellosis-free status is the long-term objective; in order to protect public health and facilitate the export of live animals and products of animal origin to the European market (Ministry, 2019b).

## Control programmes for Cattle Diseases in Albania with no or limited regulations at EU level

Out of 24 non-EU-regulated cattle diseases, 11 of them are endemic in Albania (Supplementary Table 3). This manuscript describes the status and CPs of bovine herpesvirus 1, bovine viral diarrhea, enzootic bovine leucosis, and anthrax. In addition, further information is provided regarding the status of Johne’s disease, leptospirosis, bluetongue, neosporosis, Q-fever.

## Anthrax

Anthrax is an endemic disease in Albania. The number of anthrax cases varies between years, (i.e. 40, 11, 19, and 7 laboratory-confirmed cases were recorded in, 2012, 2014, 2016, and 2018 respectively) (Ministry^[[3]](#footnote-3)^, 2019a). Historically, anthrax control was based on the vaccination of susceptible animals in villages in which anthrax cases occurred. Before 2011, more than one million animals have been vaccinated to prevent the spread of disease. Carcasses of animals that died of anthrax were not routinely safely disposed-off, which contributed to the continued risk of the disease. In 2011, the Albanian veterinary services adopted an amended approach for the control of the disease, and its control is based on timely reporting, the establishment of safe and accurate sampling (i.e. blood smears, nasal swabs), rapid standard diagnostic methodology (ISUV^[[4]](#footnote-4)^, 2014-2018), safe carcass disposal and preventive vaccination of animals at risk (Ministry, 2019a). This approach aimed to minimize environmental contamination and hence the risk of new animal infections. The approved strategy had a significant impact on both human and animal health. Both preventive and suppressive vaccination are applied. Preventive vaccination includes the vaccination of all susceptible animals at risk within the epidemiological unit for at least three years (since confirmed outbreak) on a compulsory basis. Suppressive vaccination is applied to all animals of all premises where a presumptive or a final diagnosis of anthrax outbreaks has been established (Ministry, 2019a).

## Infectious Bovine Rhinotracheitis and Infectious Pustule Vulvovaginitis (IBR/IPV)

Infectious Bovine Rhinotracheitis (IBR) is an infectious condition of cattle that is endemic in the Albanian cattle population (Lugaj et al, 2014). During an outbreak that occurred in 2008, specific antibodies to Bovine Herpes Virus Type 1 (BoHV) – 1 and BoHV – 4 were detected in 91.4% and 88.6%, of the herds respectively (Koleci^[[5]](#footnote-5)^ et al, 2010). Currently, there is no state or private national CP established for controlling IBR/IPV and both prevalence and incidence are poorly documented. However, in several commercial dairy herds (>10 cattle) there is a voluntary initiative to control the disease based on a vaccination programme. Both bivalent and multivalent vaccines are in use. In a few cases, intranasal vaccination is performed in farms where a clinical BoHV-1 outbreak has been detected. There are no conditions in place for granting an IBR/IPV health status.

Farmers are not very motivated to participate in IBR/IPV control because of the additional costs involved, the lack of rewards for a higher herd health status and a lack of understanding/awareness of the costs of the disease.

## Enzootic bovine leucosis (EBL)

Enzootic bovine leucosis is a viral infectious disease that used to be an issue for the cattle industry in Albania; however, it has been rarely reported in the last two decades. The competent authority has outlined control measures (according to OIE guidance) in case of potential outbreaks, however, there is no active surveillance programme in place.

Passive surveillance is conducted in the field, i.e., veterinarians must report suspected cases. The animal is considered suspicious if it has enlarged lymph nodes or when the animal has marked lymphocytosis. In such cases (in the last decades only a few cases were reported), the official veterinarian conducts an epidemiological investigation and orders the testing (Agar Gel Immune Diffusion is available) of all animals in the holding. The positive animals are removed, and the herd is retested periodically until it has two subsequent negative test results.

## Bovine viral diarrhea (BVD)

Currently, in Albania, there is no national BVD CP in place, however, presence of suspected clinical cases and positive serological results are reported in dairy cattle. A cross-sectional study conducted in the dairy herds of three districts revealed an overall serological herd prevalence of 28.8% (Koleci et al^[[6]](#footnote-6)^, 2014). A private CP based on vaccination is applied in some commercial dairy farms (>20 cattle). This CP is based on voluntary agreements between farmers and their associated veterinarians. Farmers sometimes do not apply for a rigorous vaccination programme and fail to control the infection. This programme uses a multivalent vaccine that includes two BVDV types (both BVDV1 and BVDV2) and some of the other common respiratory viral pathogens of cattle. The farmers involved in the CP can keep their replacement animals or buy them from EU - countries. The CP does not include other control measures such as detection and removal of persistent infection (PI) animals. No certified health statuses are given and therefore no farms are able to obtain a BVD-free status.

## Bluetongue (BT)

Bluetongue is a vector-borne disease caused by the bluetongue virus. It is typically a sheep disease; however, some strains may affect cattle. The last bluetongue outbreaks were reported in October 2020 (OIE, 2020). Previously it was found that Bluetongue virus serotype 9 in 2004 (BTV – 9) (Ventura et al, 2004) and BTV – 4 had been circulating in Albanian sheep, goats, and cattle since 2014 (Dedolli et al, 2017). The first incursion of bluetongue in Albania occurred in 1998 – 1999. A study carried out in 2002 found that the highest seroprevalence region (at animal level) was Tirana with 61% in cattle and 20% in small ruminants. Sero-neutralization testing confirmed serotype 9 (Ventura et al, 2004). Sero-neutralization testing confirmed serotype 4 for the first time in late summer 2014 in south-east Albania bordering with Greece and North Macedonia, where clinical cases were recorded in sheep, goats, and cattle. Sheep were the most affected among the susceptible species with 31,671 cases (apparent morbidity 14.2%) and 7,410 deaths (case fatality rate 23.4%) (Dedolli et al, 2017).

Although there are vaccines available against BTV-4, they are not approved for use in Albania. In addition, no data on Culicoides species, their distribution, and population dynamics are available.

## Paratuberculosis (Johne’s disease)

Paratuberculosis is a chronic bacterial disease that affects cattle, small ruminants and there are concerns that it may be zoonotic, though this remains an issue of debate and controversy. The first clinical cases, of Johne’s disease in Albania, were reported in 1970 (Keçi, 2014). A longitudinal study carried out in five Albanian dairy cattle herds showed that 24.7% of animals tested positive for the presence of specific antibodies to *Mycobacterium avium* subsp. *paratuberculosis*. Clinical and serological evidence indicated that paratuberculosis is endemic in dairy cattle (ISUV, 2018, Keçi & Koleci, 2014). There is no CP in place to deal with confirmed positive herds. Clinical suspected animals and animals with positive serological test results are generally removed from herds, but no other control measures are applied.

## Bovine genital campylobacteriosis

There are no data available regarding the prevalence of bovine genital campylobacteriosis and its role on the cattle reproduction tract in Albania. There is no CP in place either in cows or bulls.

## Q fever

Q fever is a notifiable disease in Albania and occurs only sporadically. Results of a serological study showed that specific IgG against *Coxiella burnetii* was present in 13.3% of sheep, 17.7 % of goats, and 4% of cattle (Rapti et al, 2011). Another study found that 13.51% of sheep, 9.79% of goats, and 16.43% of cattle tested positive for specific antibodies to *Coxiella burnetii* (Bicoku et al, 2013). These differences may be related to different study designs and may reflect the epidemiological situation of particular regions involved in each study. Currently, there are no control measures in place, either at the national or local level.

## Neosporosis

There are no published data on bovine neosporosis, however, *Neospora caninum* antibodies were detected in dog sera (Hamel et al, 2016). Unpublished data (Koleci Xh. personal communication) indicate that *N. caninum* antibodies were present in 30 out of 188 cows (16%) that had aborted and had also been tested negative for brucellosis. There is no control measure in place.

## Leptospirosis

Leptospirosis is an endemic but neglected disease that affects both animals (including cattle) and humans in Albania. In a cross-sectional study conducted in 2012 on 1026 cattle, the overall animal prevalence of leptospirosis was found to be 9.5% and specific antibodies for 8 different serovars of Leptospira were identified. The most frequent was *L*. hardjo, followed in turn by *L*. icterohaemorrhagiae, *L*. gropotyphosa, *L*. Pomona, *L*. Canicola, *L*. Bratislava, *L*. Copenhagen, and *L.* Tarasovi, (Alla et al, 2016). No leptospirosis CP is in place.

## Mastitis caused by *Staphylococcus aureus* and *Streptococcus agalactiae*

Mastitis is prevalent in Albanian dairy cattle. *Staphylococcus aureus* and *Streptococcus agalctiae* are commonly isolated and responsible for a substantial portion of subclinical and clinical mastitis cases. There is no CP in place to monitor subclinical mastitis in Albania. Most Albanian isolates from clinical mastitis are identified as *S. aureus* (ISUV, 2014 – 2018).

# References

1. Alla, L., Bino, S. Brucellosis, frequency of occurrence, number of cases reported, and incidence (cases per 100.000 inhabitants) for periods 2010 - 2013. (2014) Nr 2:27-36 www.ishp.gov.al/wpcontent/uploads/2015/.../buletini-3-2015.pdf
2. Alla, L., Bino, S. Incidence of zoonotic disease (Anthrax and Brucellosis) in 2015 in Albania. Public Health Institute bulletin. (2016) Nr 3:27-36 www.ishp.gov.al/wp-content/ uploads/.../ buletini/buletini_2_2014.pdf
3. Alla, L., Keçi, R., Koleci, Xh. Serologic Study on Leptospirosis Infection in Cattle in Main Albanian Coastline Districts. Int. J. Curr. Microbiol. App. Sci. (2016). 5(10): 10-15. doi: http://dx.doi.org/10.20546/ijcmas.2016.510.002
4. Bicoku Y, Saiti I, Lugaj A, Korro K, Berxholi K. Serological Survey of Q Fever in Small Ruminants and Cattle in Five Regions of Albania: An update. Journal of Animal and Veterinary Advances. (2013). 12: 402-405.
5. Dedolli K, Koni A, Keçi R, Koleci Xh. Clinical and serological study on bluetongue virus serotype 4 in cattle in Albania. Animal Husbandry, Dairy and Veterinary Science Journal. (2017). 1-4 doi: 10.15761/ahdvs.1000105
6. EFSA (European Food Safety Authority). Scientific report on lumpy skin disease: I. Data collection and analysis. EFSA Journal. (2017); 15(4): 4773, 54 pp. doi:10.2903/j.efsa. 2017.4773
7. EFSA (European Food Safety Authority). Lumpy skin disease II. Data collection and analysis. EFSA J. (2018);16(2):e05176. Published 2018 Feb 19. doi:10.2903/j.efsa.2018.5176
8. Hamel D, Shukullari E, Rapti Dh, Silaghi C, Pfister K, Rehbein S. Parasites and vector-borne pathogens in client-owned dogs in Albania. Blood pathogens and seroprevalences of parasitic and other infectious agents. Parasitol. Res. (2016) 115, 489–499 doi: 10.1007/s00436-015-4765-8
9. Keçi R, Koleci Xh. Epidemiology of John’s Disease, a Review. Albanian Journal of Agricultural Science. (2014). 13 (3):1-8.
10. Koni A, Juma A, Morini M, Nardelli S, Connor R, Koleci X. Assessment of an ELISA method to support surveillance of bovine tuberculosis in Albania. Ir. Vet. J. 2016;69(1):11. [PMC free article] [PubMed] [Google Scholar]
11. Mercier A, Arsevska E, Bournez L, Bronner A, Calavas D, Cauchard J, Falala S, Caufour P, Tisseuil C, Lefrançois T, Lancelot R. Spread rate of lumpy skin disease in the Balkans, 2015-2016. Transbound Emerg Dis. 2018 Feb;65(1):240-243. doi: 10.1111/tbed.12624. Epub 2017 Feb 26. PMID: 28239954.
12. Mersinaj K, Juma A, Haxha L, Shehu F, Koleci Xh. An overview of brucellosis control in Albania during 1925 – 2012. Albanian Journal of Agricultural Science. (2013). (Special issue), 53-56.
13. Mutolo M.J, Jenny L.L, Buszek A.R, Fenton T.W, Foran D.R. Osteological and molecular identification of Brucellosis in ancient Butrint, Albania. American Journal of Physical Anthropology. (2012). 147, 254-263. doi: 10.1002/ajpa.21643
14. OIE. Bluetongue, Albania. (2020) <https://www.oie.int>
15. Quinn PJ, Markey BK, Leonard FC, Hartigan P, Fanning S, FitzPatrick ES Brucella species in Veterinary Microbiology and Microbial Disease Textbook second edition. Wiley-Blackwell (2011). 334-339.
16. Quinn PJ, Markey BK, Leonard FC, Hartigan P, Fanning S, FitzPatrick ES (2011) Mycobacterium species in Veterinary Microbiology and Microbial Disease Textbook second edition. Wiley-Blackwell (2011).161-176.
17. Rapti Dh, Postoli R, Bizhga B, Zalla P, Koleci Xh, Selmani R. Preliminary data on the serological investigation to Q fever, Lyme disease, and echinococcus in Albania. Macedonian Journal of Animal Science (2011) 1(1), pp. 21–25
18. Ventura DM, Tittarelli M, Semproni G, Bonfini B, Savini G, Conte A, Lika A. Serological surveillance of bluetongue virus in cattle, sheep, and goats in Albania. Vet Ital. (2004). 40:101–104

1. Ministry of Agricultural and rural development of Albania. Control of Bovine Tuberculosis in Albania. (2019c). <https://bujqesia.gov.al/wp-content/uploads/2019/11/> [↑](#footnote-ref-1)
2. Ministry of Agricultural and Rural Development of Albania. Control of brucellosis in livestock in Albania. (2019a). <https://bujqesia.gov.al/wp-content/uploads/2019/11/> [↑](#footnote-ref-2)
3. Ministry of Agricultural and Rural Development of Albania. Control of anthrax in livestock in Albania. (2019a). <https://bujqesia.gov.al/wp-content/uploads/2019/11/> [↑](#footnote-ref-3)
4. Food Safety and Veterinary Institute (ISUV). Annual report of Food Safety and Veterinary Institute (2014 -2018) [↑](#footnote-ref-4)
5. Koleci, Xh., Lika, A, Hala, L., Ramolli, L. Outbreak control of infectious bovine rhinotracheitis in an Albania dairy cattle. The Act of Congress, XVIII International Congress of Mediterranean Federation of Health and Production of ruminants. (2010). 418 – 422 [↑](#footnote-ref-5)
6. Koleci Xh, Koni A, Postoli R, Rapti Dh, Keçi R. Preliminary results on bovine viral diarrhea antibody on cattle population in Albania, based on ELISA test results. Book of proceedings, International Scientific Symposium of Agriculture and Veterinary. (2014). (ISSAV, October 1 – 2, 2014, University of Prishtina, Kosovo) [↑](#footnote-ref-6)
