# Supplementary material for: An Overview of Current Approaches and Challenges to the Control of Endemic Infectious Cattle Diseases in Albania
Source: Front Vet Sci. 2021 Jul 14;8:671873. doi: 10.3389/fvets.2021.671873 (PMC8317491; doi:10.3389/fvets.2021.671873)
Supplement: Supplementary file 2 [file Data_Sheet_2.pdf]

***Supplementary Table 1** – Cattle density on agricultural land in Albanian regions (extracted from INSTAT, 2019)*

**Region**	**Number of cattle**	**Agricultural land km^2**	**Cattle density agricultural area**
Berat	22,477	602	37
Dibër	35,500	752	47
Durrës	23,100	431	54
Elbasan	43,125	1205	36
Fier	67,880	1335	51
Gjirokastër	18,800	1091	17
Korçë	39,377	1480	27
Kukës	25,390	551	46
Lezhe	33,050	468	71
Shkodër	40,155	734	55
Tiranë	40,137	670	60
Vlorë	26,618	981	27
**Total**	415,609	10300	40

Supplementary Table 2 - *Cattle farm structure in Albania*

**Nr**	Region	Total cattle farm	Farm with cattle only	Mix farm (cattle + sheep and goats)	Cows	Cows /herd	% cattle farm only
**1**	Berat	6617	3622	2995	16,435	2.5	54.7
**2**	Diber	12175	9066	3109	27,200	2.2	74.5
**3**	Durres	7680	6697	983	18,100	2.4	87.2
**4**	Elbasan	18024	11090	6934	33,900	1.9	61.5
**5**	Fier	16913	12286	4627	46,755	2.8	72.6
**6**	Gjirokaster	3418	1721	1697	13,020	3.8	50.4
**7**	Korce	14754	9745	5009	31,590	2.1	66.0
**8**	Kukes	7984	6779	1205	22,500	2.8	84.9
**9**	Lezhe	7319	6187	1132	22,770	3.1	84.5
**10**	Shkoder	9481	6561	2920	30,150	3.2	69.2
**11**	Vlore	4879	2812	2067	31,425	6.4	57.6
**12**	Tirane	12236	9178	3058	22,581	1.8	75.0
**13**	**Total**	**121480**	**85744**	**35736**	**316,426**	**2.6**	**70.6**

This is a provisional file, not the final typeset article

Supplementary Table 3 *– List of non–EU regulated cattle disease and their status in Albania*

Nr	Disease	Present/Absent /Unknown	CP in place or not	Active/Passive surveillance
1	Infectious Bovine Rhinotracheitis (IBR)	Present in dairy herds	Regional voluntary CP	Passive
2	Enzootic Bovine Leukosis (EBL)	Present Sporadic	No CP	Passive
3	Bovine Viral Diarrhea (BVD)	Present, prevalent in dairy herds	Regional voluntary CP	Passive
4	Bluetongue (BTV-4)	Present, prevalent in dairy herds	No CP	Passive
5	Paratuberculosis	Present, prevalent in dairy herds	No CP.	Passive
6	Bovine Genital Campylobacteriosis	Unknown	No CP	No
7	Anthrax	Present	National CP	Passive
8	Trichomonosis	Absent	No CP	No
9	Salmonella	Unknown	No CP	No
10	Q-fever	Present	No CP	No
11	Neosporosis	Present	No CP	No
12	Leptospirosis	Present	No CP	Passive
13	Epizootic Hemorrhagic Disease	Absent	No CP	No
14	Liver fluke	Present	No CP	Passive
15	*Staphylococcus aureus*	Present	No CP	Passive
16	*Mycoplasma bovis*	Unknown	No CP	No
17	Surra	Absent	No CP	No
18	Aujeszky’s Disease	Absent	No CP	No
19	*Mycoplasma mycoides*	Absent	No CP	No
20	*Trichophyton verrucosum*	Present	No CP	No
21	Bovine Coronavirus	Unknown	No CP	No
22	Bovine Respiratory Syncytial Virus	Unknown	No CP	No
23	Bovine digital dermatitis	Unknown	No CP	No
24	*Streptococcus agalactiae*	Present	No CP	Passive
